# Draft Whole-Genome Sequences of 14 Vibrio parahaemolyticus Clinical Isolates with an Ambiguous K Serogroup

**DOI:** 10.1128/genomeA.00217-15

**Published:** 2015-04-02

**Authors:** J. Ronholm, N. Petronella, R. Kenwell, S. Banerjee

**Affiliations:** a Microbiology Research Division, Bureau of Microbial Hazards, Food Directorate, Health Products and Food Branch, Health Canada, Ottawa, Ontario, Canada; b Biostatistics and Modelling Division, Bureau of Food Surveillance and Science Integration, Food Directorate, Health Products and Food Branch, Health Canada, Ottawa, Ontario, Canada

## Abstract

Vibrio parahaemolyticus is a bacterial pathogen responsible for mild to severe gastroenteritis, wound infections, and septicemia resulting from the ingestion or handling of raw or undercooked contaminated seafood. Here, we report the draft whole-genome sequences and annotations of 14 Canadian V. parahaemolyticus clinical isolates that were serologically identified as K group II using polyvalent antisera but were not specifically K serogrouped using monovalent antisera.

## GENOME ANNOUNCEMENT

Vibrio parahaemolyticus is a halophilic marine bacterium that is widely distributed in temperate estuaries and is one of several etiological agents of human vibriosis. Since 2000, there has been an increasing prevalence of V. parahaemolyticus infections in Canada (1). However, the true incidence of infection is likely underestimated, due to a lack of awareness of the disease and its self-limiting nature. For effective V. parahaemolyticus epidemiological surveillance, including source attribution, strain delineation is necessary. Serology, the classic method of V. parahaemolyticus surveillance, has been unreliable in tracking the spread of outbreak-associated clonal complexes (CC), since several serovariants can simultaneously be associated with illness (2). In particular, two serotypes (O4:K12 and O12:K12) of CC36 are responsible for outbreaks associated with the consumption of raw oysters harvested on the North American Pacific coast; the two serotypes are descended from a common sequence type 36 (ST36) ancestor (3). So far, the genomic sequence of only one strain belonging to the V. parahaemolyticus CC36 (serotype O4:K12) has been published (3).

Between 2000 and 2009, several V. parahaemolyticus clinical isolates originating from provincial public health laboratories along the Pacific coast were submitted to the National Microbiology Laboratory (Public Health Agency of Canada), British Columbia Centre for Disease Control (BCCDC), and the Bureau of Microbial Hazards (BMH) (Health Canada). Twenty-six of these isolates were identified as ST36 and O4, indicating inclusion in CC36, but only weakly agglutinated with the polyvalent antiserum K group II and failed to agglutinate with any of the seven associated monovalent antisera (K agglutinins 9, 10, 11, 12, 13, 15, and 17) (4). Each of these 26 isolates was positive for both the *tdh* and *trh* virulence markers (4). Since K group II isolates are a prevalent cause of Canadian illness, genome sequencing was undertaken as an approach to further investigate the genetics underlying ambiguous serological classification.

Briefly, sequencing was performed as described by Petronella et al. (5) and Pightling and Pagotto (6). Sequencing libraries were prepared from DNA extracted using the Maxwell 16 SEV cell DNA purification kit (Promega, Madison, WI). The short-read sequence data were generated by preparing a paired-end library with the Nextera XT DNA sample preparation kit (Illumina, San Diego, CA) and sequencing the library on a MiSeq benchtop sequencer (Illumina) for 500 cycles. The reads were assembled *de novo* into high-quality draft genomes with SPAdes version 3.1.1 (7), utilizing the MismatchCorrector tool, and error correction was performed with BayesHammer (8). This resulted in nonoverlapping contiguous sequences for each genome (Table 1), each of which had a total G+C content of 45%. The gene predictions and annotations were performed by the National Center for Biotechnology Information (NCBI) Prokaryotic Genome Annotation Pipeline (PGAP) (9).

**TABLE 1:** Sequencing and annotation results of 14 V. parahaemolyticus KII clinical isolates

Strain identification no.	Biosample	Accession no.	Genome coverage (%)	Genome size (bp)	No. of nonoverlapping contigs	No. of ORFs^*a*^	No. of tRNAs	No. of rRNAs
04-1290	SAMN03287716	JXVK00000000	111.05	5,143,304	97	4,767	122	27
09-3216	SAMN03287714	JXVJ00000000	99.81	5,100,021	78	4,715	125	37
10-4293	SAMN03287764	JXVA00000000	50.09	5,202,165	58	4,841	123	30
10-4303	SAMN03287766	JXUY00000000	55.97	5,106,734	52	4,708	117	29
10-7197	SAMN03287767	JXUX00000000	30.68	5,091,435	56	4,684	116	26
10-4298	SAMN03287765	JXUZ00000000	44.87	5,233,510	76	4,829	118	29
10-4288	SAMN03287763	JXVB00000000	70.12	5,109,523	61	4,717	128	28
10-4274	SAMN03287762	JXVC00000000	73.38	5,115,101	96	4,751	120	26
10-4241	SAMN03287715	JXVI00000000	43.68	5,104,503	57	4,719	128	28
10-4242	SAMN03287757	JXVH00000000	54.82	5,126,748	74	4,758	124	29
10-4245	SAMN03287758	JXVG00000000	66.30	5,097,053	70	4,697	121	28
10-4246	SAMN03287759	JXVF00000000	79.87	5,098,357	74	4,704	124	27
10-4247	SAMN03287760	JXVE00000000	106.56	5,124,180	84	4,745	124	29
10-4248	SAMN03287761	JXVD00000000	101.36	5,112,922	117	4,737	122	37

a ORFs, open reading frames.

### Nucleotide sequence accession numbers.

These nucleotide sequences have been deposited at DDBJ/EMBL/GenBank as BioProject PRJNA272927 under the accession numbers provided in Table 1.
